# Response surface methodology for optimization of nitrocellulose preparation from nata de coco bacterial cellulose for propellant formulation

**DOI:** 10.1016/j.heliyon.2024.e25993

**Published:** 2024-02-07

**Authors:** Nursyafiqah Jori Roslan, Siti Hasnawati Jamal, Jahwarhar Izuan Abdul Rashid, Mohd Nor Faiz Norrrahim, Keat Khim Ong, Wan Md Zin Wan Yunus

**Affiliations:** a Department of Defence Science, Faculty of Defence Science and Technology, National Defence University of Malaysia, 57000, Kuala Lumpur, Malaysia; b Centre for Tropicalization, Defence Research Institute, National Defence University of Malaysia, 57000, Kuala Lumpur, Malaysia; c Department of Chemistry and Biology, Centre for Defence Foundation Studies, National Defence University of Malaysia, 57000, Kuala Lumpur, Malaysia; d Research Center for Chemical Defence, Defence Research Institute, National Defence University of Malaysia, 57000, Kuala Lumpur, Malaysia

**Keywords:** Bacterial cellulose, Response surface methodology (RSM), Nitrocellulose, Cellulose

## Abstract

Nitrocellulose (NC) has garnered significant interest among researchers due to its versatile applications, contingent upon the degree of nitration that modifies the cellulose structure. For instance, NC with a high nitrogen content, exceeding 12.5%, finds utility as a key ingredient in propellant formulations, while variants with lower nitrogen content prove suitable for a range of other applications, including the formulation of printing inks, varnishes, and coatings. This communication aims to present the outcomes of our efforts to optimize the nitration reaction of bacterial cellulose to produce high-nitrogen-content NC, employing the response surface methodology (RSM). Our investigation delves into the influence of the mole ratio of sulfuric and nitric acids, reaction temperature, and nitration duration on the nitrogen content of the resultant products. Utilizing a central composite design (CCD), we identified the optimal conditions for NC synthesis. Analysis of variance (ANOVA) underscored the substantial impact of these reaction conditions on the percentage of nitrogen content (%N) yield. By implementing the predicted optimal conditions—namely, a H_2_SO_4_:HNO_3_ mole ratio of 3:1, a reaction temperature of 35 °C, and a reaction period of 22 min—we successfully produced NC with a nitrogen content of 12.64%. Characterization of these products encompassed elemental analysis, Fourier transform infrared (FTIR) spectroscopy, X-ray diffraction (XRD), thermal gravimetric analysis (TGA), and field emission scanning electron microscopy (FESEM).

## 1 Introduction

Cellulose, recognized as the Earth's most abundant biopolymer, constitutes a fundamental component in plant cell walls. Moreover, it can also be found in certain fungi, bacteria, animals, and to a lesser extent, in tunicates (small, sessile marine animals) [[1], [2], [3]]. Numerous plants, including oil palm [[4], [5], [6], [7]], pineapple [8], giant reed [9] and kenaf [10], are significant sources of this remarkable material. Nevertheless, natural cellulose often coexists with other plant components, such as lignin, hemicellulose, pectin, and starch. Consequently, extraction becomes imperative when the need arises for pure cellulose [[11], [12], [13], [14], [15]]. The presence of hydroxyl groups in the anhydroglucose units of cellulose fosters the formation of both inter- and intramolecular hydrogen bonds, resulting in cellulose's distinctive attributes, such as chain stiffness and limited solubility in numerous solvents. These characteristics render cellulose an outstanding substitute material and filler [16].

Cellulose nitrate, often represented as ([C_6_H_7_O_2_(OH)_3-x_ (ONO_2_)_x_]_n_ where monomers are linked via β (1 → 4) bonds, is commonly known as nitrocellulose (NC). This compound is obtained through the nitration of hydroxyl groups, transforming them into nitro moieties [[17], [18], [19]]. NC serves as a crucial component in the manufacturing of various industrial products. Table 1 provides a comprehensive overview of cellulose sources that have been employed to produce NC, along with their respective proposed applications.

**Table 1:** Cellulose sources to produce NC and its proposed applications.

No.	Cellulose Sources	Applications	Reference
1	*Nata de coco*	Propellant formulation	[20]
2	Non-woody feedstock of *miscanthus*	Film, membrane, lacquer, liquid bandage, artificial silk and skin	[21]
3	Mixture of kraft pulps of *pinus sp.* and e*ucalyptus sp.*	Films	[22]
4	*Acacia mangium*	Films, wood coatings, nail lacquer, automotive paint and leather finish	[23]
5	Rhizophora, oil palm empty fruit bunch and kenaf fibres	Propellant grade NC	[24]

The application of NC is contingent upon its nitrogen content (%N) or degree of nitration. When the nitrogen content falls below 12.5%, NC proves suitable for the preparation of paint, lacquer, cosmetics, varnishes, and inks. Conversely, when the nitrogen content exceeds 12.5%, NC becomes a valuable component in propellant formulations used for explosives, dynamites, smokeless gunpowder, and rocket propellants [25,26]. The assessment of NC's performance as an energetic material often centers around its thermal decomposition characteristics, including decomposition temperature, activation energy, and the process of reaction decomposition.

Recently Tarchoun et al. [27] reported interesting developments in the field of high-energy dense cellulose. Their research delved into the valorization of various lignocellulosic biomasses, including giant reed, palm fronds, and esparto grass, with the aim of transforming them into NC possessing higher nitrogen content than conventionally produced NC. Additionally, they directed their efforts toward the development of functionalized cellulose and nanostructured cellulose for NC production. Among their notable achievements was the synthesis of azidodeoxy-microcrystalline cellulose nitrate, which exhibited an impressive nitrogen content of 22.75% and demonstrated superior detonation velocity compared to traditional NC [28]. Furthermore, their research unveiled a novel class of energetic polymers, rich in cellulose and microcrystalline cellulose, functionalized with compounds such as tetrazole-acetate, carbamate nitrate, nitrate ester, nitramine, and sulfonitric acids. These reactions proved effective in producing NC with elevated nitrogen content, excellent thermal stability, favorable detonation properties, and reduced sensitivity characteristics compared to conventional NC. As a result, these findings pave the way for the design of a new generation of energetic nanostructured cellulosic biopolymers for application in advanced composite explosives and solid propellants.

The search for new potential sources of cellulose is an ongoing research activity in the synthesis of NC. Bacterial cellulose (BC) has garnered significant attention due to its distinctive characteristics, which encompass robust tensile strength, an ultrafine structure, a substantial specific surface area, and a well-defined fibrous network structure [11]. Notably, BC is primarily composed of cellulose rather than dextran [29]. In recent years, research and development endeavors centered around BC have gained momentum, emphasizing the growing significance of this emerging biopolymer. BC has exhibited exceptional performance across various applications, including audio membranes [3], medical equipment [30] and temporary pellicle skin [31].

The nitration of BC is a widely employed method for chemically modifying cellulose to produce NC suitable for a diverse range of applications. Typically, this process involves the use of nitric-sulfuric acid mixtures for BC nitration [32,33]. Response Surface Methodology (RSM), which stands for Response Surface Methodology, is a statistical tool used to design experiments and determine accurate models that relate input parameters to output variables. It utilizes the optimization capabilities of Central Composite Design (CCD) to achieve the desired response. RSM, a statistical tool, is utilized to design experiments and establish precise models that link input parameters to output variables. RSM harnesses the optimization capabilities of CCD to achieve the desired response. Notably, RSM has proven advantageous not only in industrial settings but also in various fields such as biology, medicine, automobiles, and aviation [34]. In this research, RSM is employed to investigate the relationships between parameters and to optimize NC production. The three primary parameters significantly influencing nitrocellulose production are the composition of H_2_SO_4_ and HNO_3_, reaction temperature, and reaction time. The conventional one-factor-at-a-time (OFAT) approach is often employed to study parameter-product yield relationships. However, this method has limitations as it necessitates numerous experiments and is time-consuming [35]. Furthermore, OFAT fails to elucidate the intricate interplay between each parameter and nitrocellulose production yield, as well as lacking the capacity to optimize the synthesis process. Therefore, we propose the use of RSM for this research, as it offers a statistical method to analyze parameter relationships using analysis of variance (ANOVA) [36]. The products derived from this study underwent characterization via various techniques, including elemental analysis, Fourier Transform Infrared (FTIR) spectroscopy, field-effect electron microscopy (FESEM), thermogravimetric analysis (TGA), and X-ray diffraction (XRD).

## 2 Materials and methods

### 2.1 Materials

*Nata de coco*, in the form of 1 cm × 1 cm x 1 cm cubes, was obtained from Nandong Food Industries Sdn. Bhd. Factory for this study. All the chemicals used, including 98% purity H_2_SO_4_ and 65% purity HNO_3_, were analytical grade chemicals sourced from Merck (Germany). Deionized water was prepared in the laboratory using a water deionizer.

### 2.2 Preparation of BC powder

*Nata de coco* which was supplied in an acidic condition was washed with flowing tap water to remove the acid (until pH of between 5 and 7 was obtained). The washed nata *de coco* was then rinsed three times with deionized water, dried in an oven at 80 °C until a constant weight was recorded, ground using a laboratory blender and sieved by a 125 μm sieve (AS 200, Retsch) to obtain the dry BC in a powder form.

### 2.3 Synthesis of NC

**Fig. 1:**
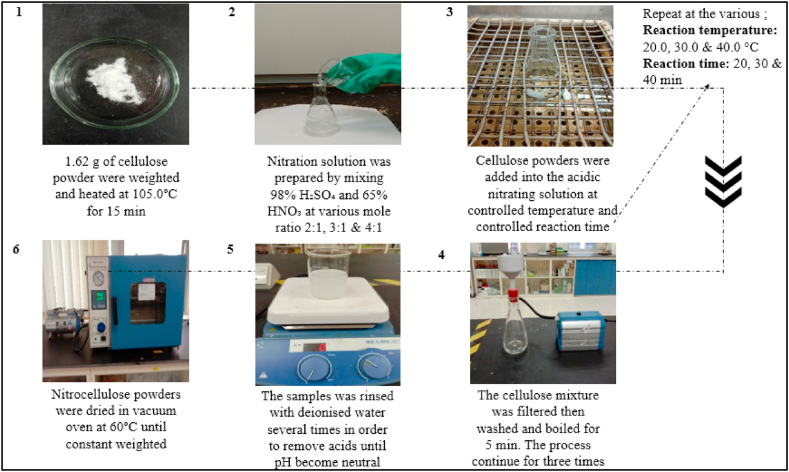
Process flow of NC synthesis.

Sulfuric-nitric acid solutions of different mole ratio were prepared by mixing the required amounts of 98% of H_2_SO_4_ and 65% of HNO_3_ solutions. The reaction temperature was controlled by placing the reaction vessel in a water bath (Wisebath, Korea). Typically, 1.62 g of BC powder which was pre-heated at 105 °C for 15 min was reacted with the nitrating solution. The product was stabilized by washing it 3 times with and soaking it for 5 min in boiling deionized water and dried it in a vacuum oven at 60 °C until a constant weight was obtained. The NC synthesis process is summarized in Fig. 1.

### 2.4 Characterization

#### 2.4.1 Fourier transform infrared spectroscopy

The infrared spectra of BC and NC were obtained using an FTIR spectra 2000 spectrophotometer (PerkinElmer) equipped with a diamond attenuated total reflectance (ATR) accessory. The spectra were recorded over the range of 4000–500 cm^−1^ with a spectral resolution of 4 cm^−1^.

#### 2.4.2 Elemental analysis

The nitrogen content (%N) was determined by an elemental analyzer (Vario EL III, Elementar, Hanau, Germany) of samples. The weight sample used is around 5.00 mg. The conversion of the percentage into the degree of substitution was carried out using the following equation (Eq (1) [23].

(1) $$Degree\;of\;Substitution\;\left(DS\right)=\frac{3.6\times Nitrogen\;content\;\left(\%\right)}{31.13\;-\;Nitrogen\;content\;\left(\%\right)}$$

#### 2.4.3 Field emission scanning electron microscopy (FESEM)

The morphologies of BC and NC powder samples were examined using a field emission scanning electron microscope model S–3400 N manufactured by Hitachi. Prior to observation, the samples were coated with a thin layer of gold using a sputter coater model K550× from Emitech. This gold coating helps to enhance the conductivity of the samples and improve the quality of the SEM images. The samples were then observed under the microscope at a magnification of 50,000×.

#### 2.4.4 X-ray diffraction

The XRD measurement was performed using a Bruker model D8 advance power diffractometer with Cu Kα sealed tube (λ = 1.54 Å). The sample was mounted in the sample holder and its intensity was measured for 2θ from 10 to 40° at a step size of 0.04°. The sample crystallinity was calculated using the equation as below:

$$Crl=\frac{I002-Iam}{I002}\times 100\%$$

which *I*002 is equal to 2θ = 22.9 and it refers to the peak of the crystalline portion of biomass (*i.e.*, cellulose), while *Iam* refers to the peak at about 2θ = 16.8, which corresponds to the amorphous region.

#### 2.4.5 Thermal gravimetric and differential thermogravimetric analyses

The TGA and DTG analyses were conducted using a PerkinElmer STA 8000 analyzer under nitrogen atmosphere. A sample mass of 2.0 mg was used, which were conducted in the temperature range of 50 °C with 10 °C/min heating rate.

### 2.5 Experimental design and statistical analysis

RSM, a statistical technique commonly used for optimizing various processes, was employed in this research to optimize the reaction parameters for NC production [37]. The study focused on three independent variables: (a) sulfuric acid to nitric acid mole ratio, (b) reaction temperature, and (c) reaction period.

To design the experiments, statistical software Design Expert version 6.0.6 (StatEase Inc., Minneapolis, USA) was utilized. A three-factor-three-level second-order face-centered central composite design (CCD) was chosen to investigate the effects of the parameters (sulfuric acid to nitric acid mole ratio, reaction temperature, and reaction time) on the percentage of nitrogen content in NC. The ranges of the variables and their coded values (−1, 0, 1 representing low, basal low, high) were determined based on preliminary studies and literature, and are summarized in Table 2. The arrangement of the central composite design, along with the corresponding dependent variable (percentage of nitrogen content), is presented in Table 3. Additionally, Table 3 also includes the results of the 20 experimental runs conducted to determine the actual values of nitrogen content in NC.

**Table 2:** Range of variables and their coded levels.

Independent variables	Coded value		
	−1	0	+1
(a) Sulfuric acid to nitric acid mole ratio (mol/mol)	2 : 1	3 : 1	4 : 1
(b) Temperature (◦C)	20	30	40
(c) Time (min)	20	30	40

**Table 3:** The arrangement of the central composite design.

Run	Independent variables			Dependent variables	
	A	B	C	Percentage of nitrogen content (%N)	
				Actual value	Predicted value
1	2.00	30.00	30.00	11.83	11.92
2	4.00	30.00	30.00	12.07	12.07
3	3.00	30.00	30.00	12.61	12.66
4	3.00	30.00	40.00	12.56	12.61
5	2.00	40.00	20.00	11.60	11.62
6	2.00	20.00	40.00	11.06	11.04
7	3.00	30.00	30.00	12.75	12.66
9	2.00	40.00	40.00	11.68	11.65
10	3.00	40.00	30.00	12.58	12.55
11	3.00	30.00	20.00	12.38	12.41
12	4.00	40.00	40.00	11.96	12.00
13	3.00	30.00	30.00	12.76	12.66
14	3.00	20.00	30.00	11.74	11.85
15	3.00	30.00	30.00	12.69	12.66
16	4.00	20.00	40.00	11.30	11.26
17	2.00	20.00	20.00	11.01	10.95
18	3.00	30.00	30.00	12.62	12.66
19	4.00	20.00	20.00	10.89	10.90
20	3.00	30.00	30.00	12.71	12.66

## 3 Results and discussion

### 3.1 Optimization of NC production

#### 3.1.1 Quadratic regression model for production of NC

The optimization of NC production was conducted utilizing a RSM model, a technique rooted in the principles of Design of Experiments (DOE). In this study, a second-order polynomial RSM equation (Eq. 2) was employed to forecast the percentage of nitrogen content. The prediction results are presented in Table 3, with Y representing the percentage of nitrogen content, while A, B, and C denote the mole ratio of sulfuric acid to nitric acid, reaction temperature, and reaction duration, respectively. To assess the statistical significance of the quadratic regression model and the impact of significant individual correlations, we analyzed the data using Analysis of Variance (ANOVA) as presented in Table 4. The model's adequacy was gauged by comparing actual and predicted values, and the percentage of residual error (RE) was calculated based on the response [[38], [39], [40]]. The percentage of nitrogen content obtained ranged from 10.89% to 12.76%, aligning well with values previously reported by Budaeva et al. (2019) [33].

(2) $$Y=12.66+0.073A+0.35B+0.099C-0.67A^{2}-0.46B^{2}-0.15C^{2}+0.031AB+0.069AC\;-\;0.013BC$$

**Table 4:** Analysis of variance (ANOVA) for regression model to optimize condition in the synthesis of NC.

Source	Sum of squares (SS)	Degree of freedom (DF)	Mean square (MS)	F-value	p-value	Remarks
Model	7.53	9	0.84	128.34	<0.0001	Significant
Residual	0.059	9	6.519 × 10^−3^			
Lack of fit	0.038	4	9.618 × 10^−3^	2.38	0.1836	Not significant
Pure error	0.020	5	4.040 × 10^−3^			
R-Squared	0.9923					
Adequacy Precision	0.8511					
Std. Dev	0.081					
Mean	12.04					

Std. Dev = Standard Deviation.

The results of this study indicate that the obtained model is highly significant, as evidenced by the F-test value analyzed using ANOVA with a probability value of <0.00011. This implies that there is only a 0.01% chance that a model of this magnitude could occur due to noise. Additionally, the *P*-value (probability of error value) < 0.0500 indicates that the model terms are statistically significant. A large F-value and small *P*-value suggest that the independent variables have a significant impact on the response variable.

Furthermore, the lack of fit value, which is an important consideration in this experiment, is found to be 0.1836, indicating no significant lack of fit. In this context, the significant model terms encompass A, B, C, A^2^, B^2^, C^2^, and AC. A p-value exceeding 0.1000 indicates the non-significance of model terms. In cases where numerous non-significant model terms emerge (excluding those essential for maintaining hierarchy), adjusting the parameter range may enhance the model's performance.

**Fig. 2:**
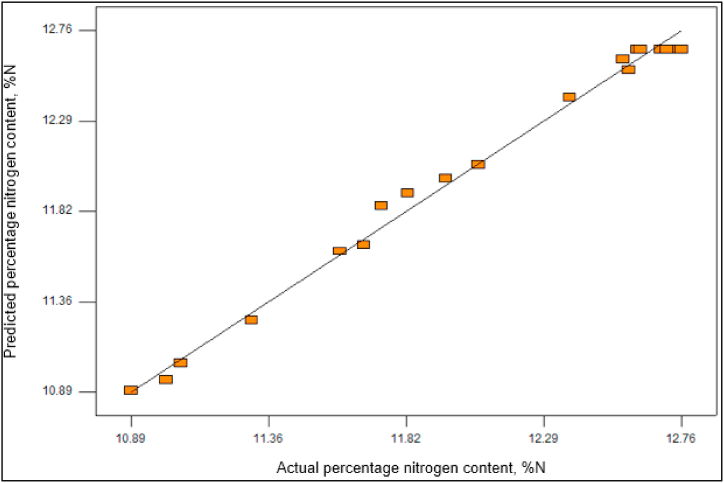
Experimental and predicted value of synthesis NC analysis of RSM.

The coefficient of determination, often denoted as R-squared (R^2^), serves as an indicator of the relationship between the dependent variable (percentage of nitrogen content) and the predicted variables. A higher R^2^ value signifies a stronger correlation between the model-predicted data and the experimental data, thereby demonstrating increased data reliability. The relationship between these variables is visually depicted in Fig. 2. In this study, an R^2^ value of 0.9923 was obtained, signifying that 99.23% of the variability in the experimental percentage of nitrogen content can be accounted for by the quadratic regression model. The ANOVA analysis further reaffirms the reliability and stability of the second-order quadratic model used for optimizing NC synthesis, as it consistently provides accurate predictions.

#### 3.1.2 Effect of mole ratio H_2_SO_4_/HNO_3_ and temperature on percentage nitrogen content

**Fig. 3:**
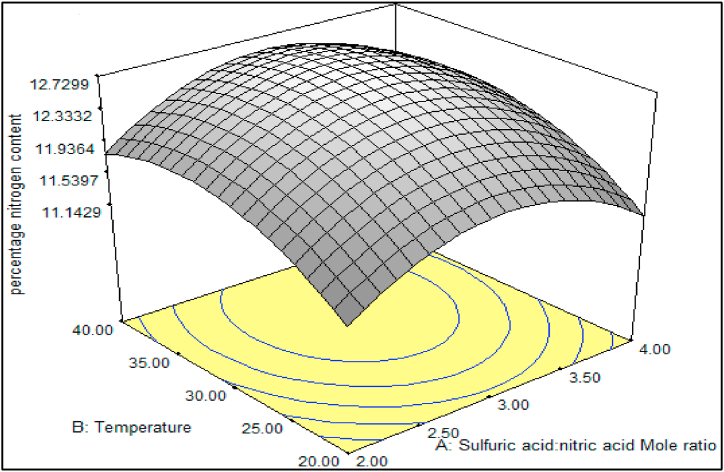
Effects of mole ratio of H_2_SO_4_/HNO_3_ and temperature at fixed reaction period of 30 min on the N content.

Fig. 3 illustrates the interaction between the mole ratio of H_2_SO_4_/HNO_3_, varying from 2:1 to 4:1, and temperature, ranging from 20 °C to 40 °C, while maintaining a fixed reaction period of 30 min. The specific focus of this analysis is on the percentage of nitrogen content (%N). As depicted in the figure, an increase in the mole ratio of H_2_SO_4_/HNO_3_ and temperature corresponds to an increase in the percentage of nitrogen content. The figure highlights that a mole ratio of H_2_SO_4_/HNO_3_ at 3:1 and a temperature ranging between 30 °C and 35 °C yield a product with a nitrogen content exceeding 12.60 %N. This percentage is deemed suitable for use as an ingredient in guns and rocket propellants [41].

#### 3.1.3 Effect of mole ratio H_2_SO_4_/HNO_3_ and reaction period on percentage of nitrogen content

**Fig. 4:**
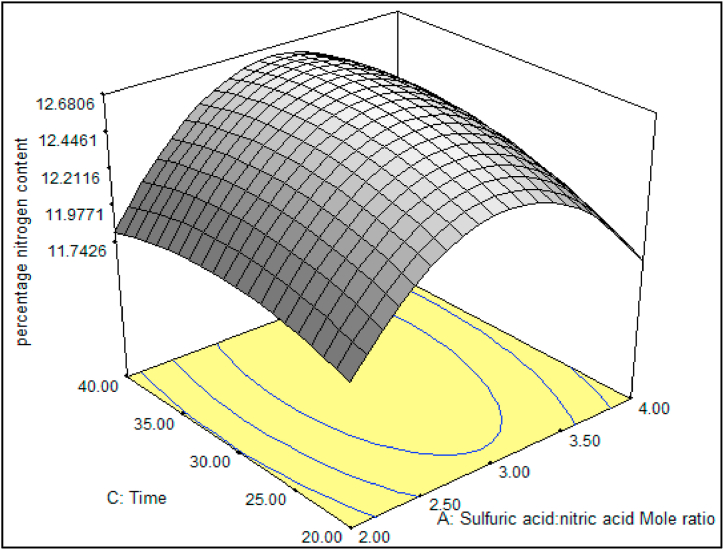
Effects of mole ratio of H_2_SO_4_/HNO_3_ and reaction period at fixed temperature of 30 °C on the N content.

Fig. 4 illustrates the interaction between varying mole ratios of sulfuric acid to nitric acid (2:1, 3:1, and 4:1) and reaction periods (ranging from 20 to 40 min) on the percentage of nitrogen content, with a fixed reaction temperature of 30 °C. The figure highlights that the optimal nitration conditions involve a mole ratio of H_2_SO_4_/HNO_3_ at 3:1 and a reaction period of 30–35 min. This observation is attributed to the increase in H_2_SO_4_ concentration, which promotes the swelling of BC, subsequently enhancing the diffusion of nitric acid into the BC molecules. These findings align with those reported in previous studies [11,42].

#### 3.1.4 Effect of temperature and reaction period on the percentage of the nitrogen content

**Fig. 5:**
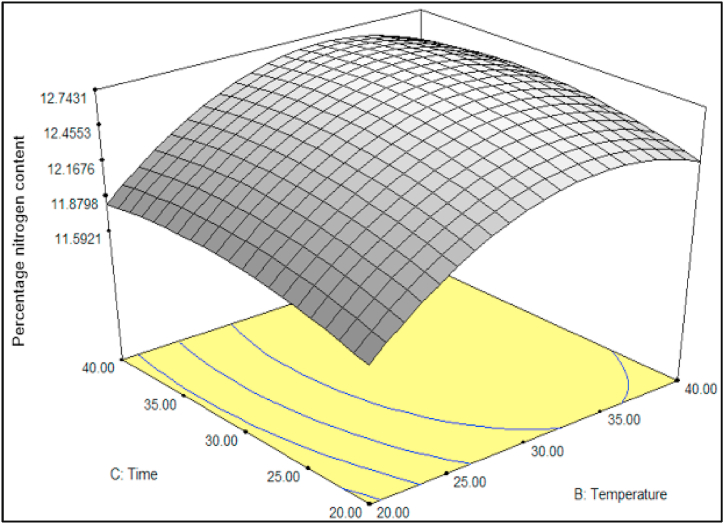
Effects of reaction period and temperature at fixed mole ratio of H_2_SO_4_/HNO_3_ 3:1 on the N content.

Fig. 5 demonstrates the effects of the interaction between reaction time (ranging from 20 to 40 min) and temperature (ranging from 20 to 40 °C), which a fixed mole ratio of H_2_SO_4_/HNO_3_ at 3:1, on the percentage of nitrogen content (%N). The figure indicates that the percentage of nitrogen content increases as the temperature and reaction time increase, reaching optimal conditions at approximately 30–35 °C and 30–35 min, respectively. Beyond these optimal conditions, the percentage of nitrogen content slightly decreases with further increases in temperature and reaction time. Fig. 5 reveals that the relationship between reaction time and temperature does not significantly impact the production of NC. This finding is similar with previous work by Sun et al. (2010) stated the insignificant influence of temperature and reaction time on the percentage of nitrogen content [11].

According to the optimization study, the model suggests that the optimal nitration reaction conditions for achieving the highest nitrogen content in NC are as follows: a mole ratio of H_2_SO_4_/HNO_3_ of 3:1 (mol/mol), a temperature of 35 °C, and a reaction time of 22 min. The predicted nitrogen content (%N) under these conditions is 12.55%. To validate the model, the experiment was repeated in triplicate using these optimum conditions. The average percentage of nitrogen content (%N) obtained from the synthesized NC was found to be 12.64% (experimental value), which is not significantly different from the predicted value. The residual standard error (RSE) of 0.71% indicates a good agreement between the actual and predicted values, as this error is less than 2% [43].

### 3.2 Fourier Transform Infrared spectroscopy

**Fig. 6:**
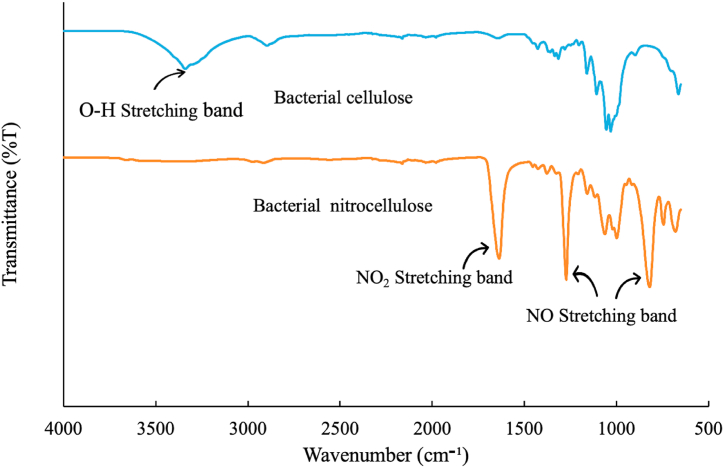
FTIR spectra of BC and NC.

FTIR spectra analysis was performed to investigate the chemical functionalities and interactions of both BC and NC. Fig. 6 displays the FTIR spectra of BC and NC. In the BC spectrum, a prominent peak at 3335 cm^−1^ corresponds to the O–H stretching band, signaling the presence of hydroxyl groups. The peak at approximately 2900 cm^−1^ signifies the C–H stretching vibration inherent to cellulose. Following the nitration process, a notable reduction in the intensity of the O–H peak around 3000 cm^−1^ is observed, indicating the substitution of hydroxyl groups by nitryl (NO_2_) groups during nitration. In the NC spectrum, three distinctive peaks affirm the presence of nitryl groups: sharp peaks located around 1629 cm^−1^, 1274 cm^−1^, and 830 cm^−1^. Furthermore, both BC and NC spectra exhibit several additional peaks, including those around 1300 cm^−1^ (C–H bending), 1400 cm^−1^ (CH_2_ bending), 1050 cm^−1^ (C–O stretching), and 1160 cm^−1^ (C–*O*–C stretching), elucidating the existence of various chemical bonds and functional groups within the samples.

### 3.3 Field emission scanning electron microscope

**Fig. 7:**
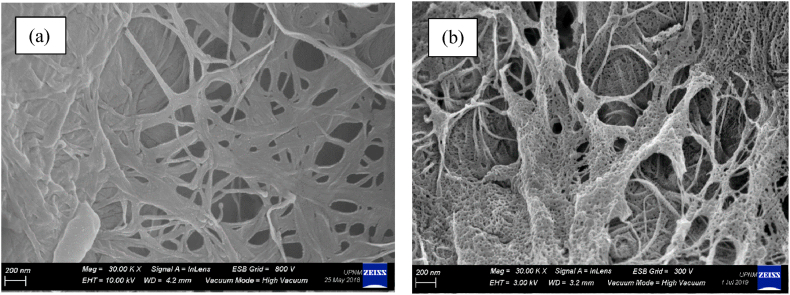
FESEM images of (a) BC and (b) NC at 30,000× magnification.

Fig. 7 displays the morphology micrographs of BC (Fig. 7(a)) and NC (Fig. 7(b)) obtained through FESEM analysis, which was used to investigate the surface morphology modifications induced by the nitration process. The micrographs were captured at a magnification of 30,000×. These micrographs reveal that the initially smooth surface of BC fibers undergoes substantial alterations after nitration, resulting in a rough and porous structure [44]. However, in addition to the roughness, the NC samples exhibit irregular rough fibrils with shorter dimensions compared to BC. These FESEM results are in line with the XRD analysis, which indicates that BC has a higher crystallinity index compared to NC. The morphological transformations depicted in the micrographs, coupled with the differences in crystallinity, can be attributed to the chemical modifications and structural transformations that transpire during the nitration process.

### 3.4 X-ray diffraction

**Fig. 8:**
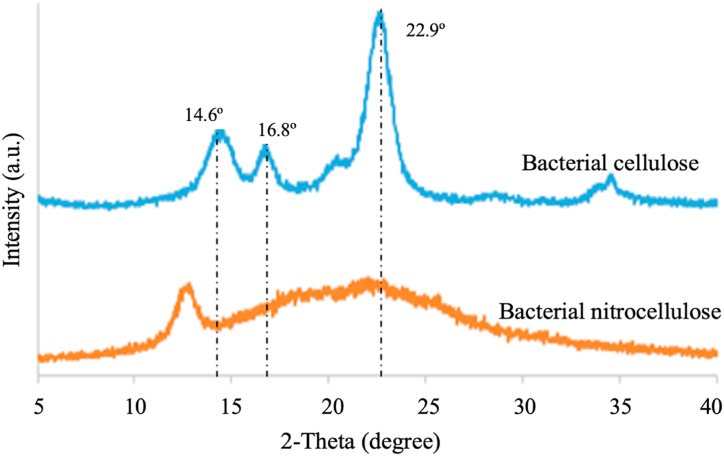
The XRD spectra of BC and NC.

The XRD analysis was conducted to explore the crystalline structure and crystallinity ratio of both BC and NC samples. Fig. 8 presents the XRD spectra of both polymers. The XRD patterns show sharper diffraction peaks at around 2θ = 14.6°, 16.8°, and 22.9°, which correspond to the cellulose structure with higher crystallinity regions. BC exhibits a higher crystallinity index (Crl) (%) of 55.09% compared to NC, which has a Crl of 12.30%. This difference can be attributed to the longer chain polymer structure of BC, where the fiber molecules are arranged in a parallel and closely packed manner, resulting in higher crystallinity. However, during the nitration process, BC's fiber molecules undergo non-uniform rearrangement, leading to decreased compactness and disruption of the robust hydrogen bonds within the cellulose structure [45].

The XRD spectrum of NC shows characteristic peaks associated with the ordered and amorphous domains of highly nitrated cellulose. This pattern is typical for NC, indicating the structural changes that occur during nitration. The lower crystallinity index of NC compared to BC is a result of the nitration process, which leads to partial debundling and fragmentation of the crystalline regions, as well as the substitution of hydroxyl groups with nitrate ester moieties. These changes significantly reduce the strong inter- and intramolecular hydrogen bonding interactions responsible for the crystalline rearrangement of cellulosic chains [27].

### 3.5 Thermal behaviour

**Fig. 9:**
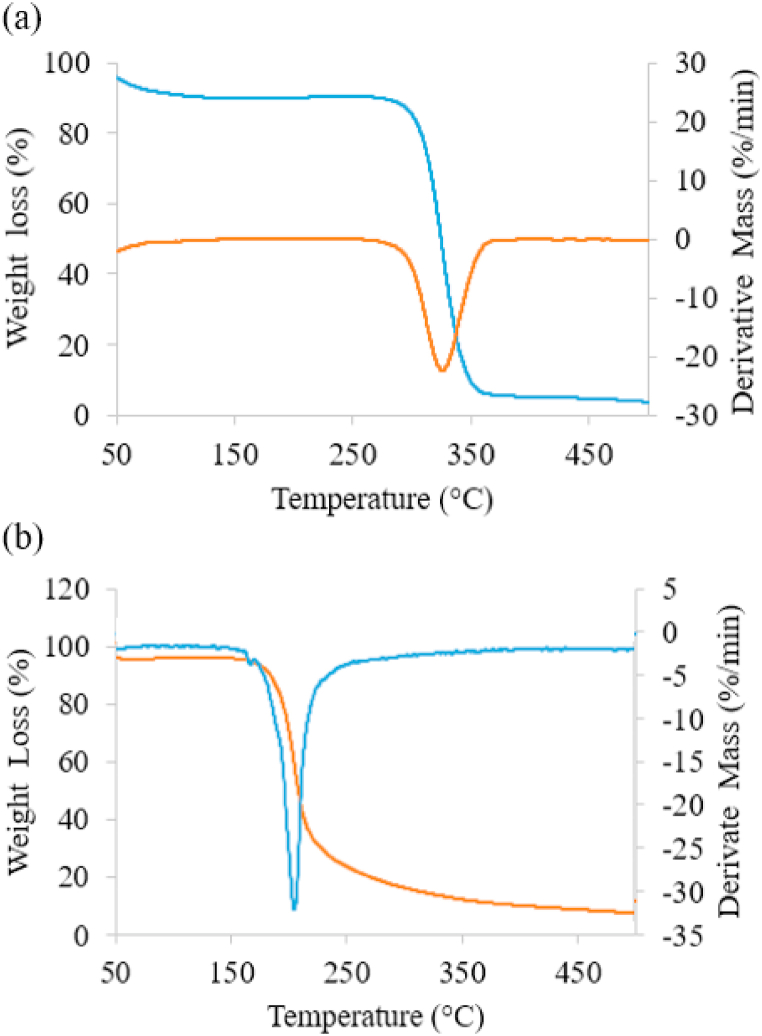
TGA/DTG thermograms of (a) BC and (b) NC.

Thermal stability and decomposition behavior of the synthesized polymer nitrates and their precursors were investigated using TGA and DTG analyses. The thermal parameter curves for BC and NC are shown in Fig. 9 (a) and (b) respectively, with two main weight loss events. The first stage, occurring in the temperature range of 70–120 °C, can be attributed to the evaporation of physically adsorbed water. BC takes more time to decompose compared to NC, mainly due to its high purity of cellulose content and higher crystallinity structure compared to NC. The decomposition of BC started at around 270 °C and continued up to approximately 350 °C. This temperature range is associated with the elimination of small molecular fragments, such as hydroxyl groups. The maximum rate of decomposition occurred around 320 °C, corresponding to polymer chain degradation and the breakdown of the pyran structure, which is a six-member cyclic structure [46]. The residual weight loss of approximately 5% shown in the DTG graph indicates that the BC content is 100% cellulose.

The TGA/DTG thermograms for NC reveal a two-stage decomposition process. The first stage occurs at around 130 °C and is attributed to the denitration reaction of NC molecules. Denitration involves a nucleophilic attack on NC, leading to the cleavage or breakage of the cellulose polymer chain. The second decomposition stage begins around 180 °C and continues up to approximately 330 °C, with the maximum rate of decomposition occurring around 200 °C. Compared to BC, NC exhibits a shorter decomposition time, which can be attributed to its reduced crystallinity structure that arranges the molecules into a more amorphous state. The higher sensitivity of NC to thermal degradation can be attributed to its high nitrogen content and the presence of nitrate functional groups. This finding aligns with the XRD results, which indicate that BC has a higher crystallinity index compared to NC. The DTG thermogram for NC also displays two-stage decompositions. The first peak is associated with the denitration process of NC molecules, as reported by Cudzilo et al. (2012) [47] and Meng & Xiao, (2018) [48]. The second peak is correlated with the autocatalytic reaction of NC, resulting in the release of nitrogen gas.

## 4 Conclusion

The RSM optimization of the nitration reaction for NC production from BC has proven to be successful, with the acid to nitric acid mole ratio, reaction temperature, and reaction period identified as the most influential factors in the synthesis process. The optimized conditions, consisting of a mole ratio of H_2_SO_4_/HNO_3_ of 3:1, temperature of 35 °C, and reaction period of 22 min, resulted in the production of NC with a nitrogen content of 12.64%. The validation of the proposed model showed good agreement with the predicted value, with a RSE of less than 0.8%. The nitrogen content obtained in this study meets the minimum concentration required for propellant formulations. Furthermore, it is anticipated that the nitrogen content can be further improved by functionalizing the BC with various compounds, such as tetrazole-acetate, carbamate nitrate, nitrate ester, nitramine, and sulfonitric acids.

## Data availability statement

Data will be made available on request.

## Additional information

No additional information is available for this paper.

## CRediT authorship contribution statement

**Nursyafiqah Jori Roslan:** Writing – original draft, Methodology, Investigation, Formal analysis, Data curation, Conceptualization. **Siti Hasnawati Jamal:** Writing – review & editing, Writing – original draft, Validation, Supervision, Project administration, Funding acquisition, Conceptualization. **Jahwarhar Izuan Abdul Rashid:** Writing – original draft, Validation, Project administration, Conceptualization. **Mohd Nor Faiz Norrrahim:** Writing – review & editing, Validation, Conceptualization. **Keat Khim Ong:** Validation, Supervision, Project administration, Data curation, Conceptualization. **Wan Md Zin Wan Yunus:** Writing – review & editing, Writing – original draft, Validation, Supervision, Project administration, Funding acquisition, Conceptualization.

## Declaration of competing interest

The authors declare that they have no known competing financial interests or personal relationships that could have appeared to influence the work reported in this paper.
